# Concurrent validity of the Ages and Stages Questionnaire and the Bayley Scales of Infant Development III in China

**DOI:** 10.1371/journal.pone.0221675

**Published:** 2019-09-05

**Authors:** Ai Yue, Qi Jiang, Biaoyue Wang, Cody Abbey, Alexis Medina, Yaojiang Shi, Scott Rozelle

**Affiliations:** 1 Center for Experimental Economics in Education (CEEE), Shaanxi Normal University, Xi’an, Shaanxi, China; 2 School of Software and Microelectronics, Peking University, Beijing, China; 3 Freeman Spogli Institute for International Studies, Stanford University, Stanford, California, United States of America; Utrecht University, NETHERLANDS

## Abstract

Choosing a valid and feasible method to measure child developmental outcomes is key to addressing developmental delays, which have been shown to be associated with high levels of unemployment, participation in crime, and teen pregnancies. However, measuring early childhood development (ECD) with multi-dimensional diagnostic tests such as the Bayley Scales of Infant and Toddler Development III (Bayley-III) can be time-consuming and expensive; therefore, parental screening tools such as the Ages and Stages Questionnaire (ASQ-3) are frequently an alternative measure of early childhood development in large-scale research. The ASQ is also becoming more frequently used as the first step to identify children at risk for developmental delays before conducting a diagnostic test to confirm. However, the effectiveness of the ASQ-3 is uncertain. In this study, we evaluate the accuracy of the ASQ-3 as a screening measure for children at risk of developmental delay in rural China by age group. To do so, we administered the Bayley-III, widely considered to be the “gold standard” of ECD diagnostic tests, to a sample of 1,831 five to twenty-four month-old children and also administered the ASQ-3 to their caregivers. We then compared the outcomes of the ASQ-3 test to those of the Bayley-III. We find that the ASQ-3 was significantly though weakly correlated with the Bayley-III and that the strength of this correlation increased with child age and was stronger when the mother was the primary caregiver (as compared to the grandmother). We also find that the sensitivity and specificity of ASQ-3 ranged widely. The overall findings suggest that the ASQ-3 may not be a very accurate screening tool for identifying developmentally delayed children, especially for children under 13 months of age or children whose primary caregiver is not the mother.

## Introduction

Development during the first two years of life is critical and has a lasting impact on a child’s health and life-long development [1]. Investing in early childhood development is highly efficient due to the rapid brain development and brain malleability during the first years of life [2]. Children with healthy development have been shown to have high levels of educational attainment, employment, and earnings later on in life [2–6], whereas poorly developed children tend to later have higher rates of unemployment, participation in crime, and teen pregnancies [4,7,8].

Previous research has explored the extent of developmental delays around the world and whether evidence-based interventions targeted at underlying causes can effectively treat developmental delays. One study showed that an alarming forty-three percent (43%) of children under 5 years of age around the world do not develop to their full potential [9]. Overall, findings in both developed and developing countries show that poor nutrition, subpar health, and the lack of interactive parenting inputs are factors that are systematically linked with ECD delays [2,5,10]. In response, much scholarship has been dedicated toward exploring the design, implementation, and effectiveness of early interventions such as teaching caregivers how to provide better nutrition and increase interactive parenting investments [11–13].

In order to administer interventions to address ECD issues, however, one needs to first identify the individuals and subpopulations that are particularly vulnerable and then measure their improvement (or deterioration) over the course of an intervention. Consequently, choosing a valid and feasible method to measure child developmental outcomes is key. As interventions are most effective in the first few years of a child’s life [14], there is an urgency to obtain measures of development for children under 3 years of age.

Several multi-dimensional diagnostic tests exist to assess the development of children across multiple aspects of development, such as cognitive skills and motor skills. Almost certainly considered by the field as the gold standard, the Bayley Scales of Infant Development (or the *Bayley-III*) is one of the most widespread scales used to measure developmental delays despite several drawbacks [15,16]. The Bayley-III is an individually administered test that assesses the development of children and young children aged 0 to 42 months. Its primary purposes are to identify children with developmental delays and to provide information for intervention planning. However, administering the Bayley-III is time consuming and requires highly trained professionals working in controlled environments. In addition, the test kits and administration fees are expensive. The Bayley-III is also unavailable in some languages, and translating the test requires specialized knowledge of the local language and culture [17].

In light of these disadvantages, scholars in the ECD field have made efforts to find feasible ways to replace or supplement the Bayley-III, including the use of parental screening tools. These tools acquire developmental information and assess development by asking caregivers to report different aspects of their child’s behavior [18,19]. The Ages and Stages Questionnaire (ASQ-3) is one of these parental screening tools. Developed in the United States, the ASQ-3 “identifies infants and toddlers between one month and 66 months of age who are at risk of a developmental delay regarding problem solving, communication, fine motor skills, gross motor skills, and personal social behavior” [18]. This type of monitoring instrument is cheaper, shorter in duration, and easier to administer than the Bayley-III.

When considering whether to use ASQ-3 as a screening tool for developmental issues in young children, one of the most important considerations is its validity. Although the concept of validity has evolved over time, it is universally accepted that validity is defined as the extent to which a concept is accurately measured in a quantitative study, which means that a valid test can convey the effect of variation in the attribute that it intends to measure [20,21]. The most recent edition of the Standards for Educational and Psychological Testing considers validity to be based on the accumulation of evidence and theory [22]. One of the most common forms of evidence is relations between a test and other variables, which has been proposed as an appropriate method for assessing the validity of psychometric tests [21]. In our study, we refer to this as concurrent validity.

Previous studies have assessed the concurrent validity of the ASQ-3 as a screening tool by comparing its results against those of the aforementioned Bayley-III. Overall, the results of such studies have varied when using the Bayley-III as the standard for comparison. Some studies found relatively high and consistent validity of the ASQ-3 for all age groups [23,24]. However, other studies found a relatively wide range of validity which inferred that ASQ-3 may not be valid enough under some circumstance [25,26].

In particular, some studies have found that the ASQ-3 is better able to assess children’s development (meaning that it more closely corresponds with the Bayley-III) when children are older rather than when they are younger [17,18,27]. For example, one study conducted in the Netherlands found that the ASQ had better sensitivity (the rate at which a screening instrument correctly identifies a developmental delay) and better specificity (the rate at which a screening instrument correctly identifies children who perform within the normal range) among children between 18–42 months old than for those between 2–16 months old [8]. Another study conducted in Colombia found that the ASQ-3 performed poorly for children under 31 months old, with generally trivial and non-significant correlations with the Bayley-III between 6 and 18 months [17].

In this paper, we examine this methodological question in the context of rural China, where there is a strong need for feasible assessment tools of developmental delays. Previous studies using Bayley-III in rural China have shown that in some villages and counties, a large portion of children are affected by developmental delays, reaching up to 48% [28,29]. There is concern that if such high rates are generalizable to all of rural China, then universal (or more widespread) screening of children may be needed. In rural China alone, approximately 17 million children are born every year. As such, if it is the case that the ASQ-3 can effectively assess the development of Chinese children, the policy implications would be substantial.

There are two main reasons why the validity of the ASQ may be different in rural China as compared to other areas previously studied. First, research has shown that rural Chinese families do not engage in interactive parenting practices, and that this has an impact on the development of rural children [29]. This is markedly different from other countries, particularly developed countries [30]. That rural Chinese parents do not engage in interactive parenting practices suggests that they may have less knowledge of the developing skills of their own children. This is a potential problem for parent-reported questionnaires such as the ASQ, which are based on the idea that the parent knows his or her child best. Second, the caregiving structure in rural Chinese families is different from other areas. Because many parents leave their children in their rural homes and travel to cities for work, a significant portion of children are “left-behind children” who are raised by grandparents [31]. Grandparents may respond very differently from parents on the ASQ; however, there is no research on how differences in primary caregivers may impact the validity of the ASQ. Therefore, there is a need for studies to examine the ASQ in the context of rural China specifically.

The overall goal of this study is to provide evidence of the validity of the ASQ-3 as a screening measure for children at risk of developmental delay in rural China. Specifically, we have four objectives. First, we compare the statistical characteristics of the Bayley-III and the ASQ-3 by age group. Second, we examine the sensitivity and specificity of the ASQ-3 as compared to the Bayley-III. Third, we study the concurrent validity between Bayley-III and ASQ-3. Fourth, we examine validity of the ASQ-3 against external characteristics that have been identified in the literature as correlated with early childhood development outcomes.

To meet these objectives, we administered the Bayley-III to a sample of 1,831 5–24 month-old children and administered the ASQ-3 to their caregivers. We then summarized the statistical characteristics of Bayley-III and ASQ-3 and examined the sensitivity and specificity of the ASQ-3 against the Bayley-III. Next, in order to assess the concurrent validity, we computed Pearson correlations for the ASQ-3 and Bayley-III. Finally, we conducted an ordinary least squares (OLS) regression between each test and a set of external characteristics including maternal education, household wealth, play activities and play materials in the home. In addition, for each of these analyses, we divided our sample by the identity of the primary caregiver (mothers versus grandmothers) in order to compare the accuracy of the ASQ-3 by primary caregiver type.

## Methods

### Ethical approval

Ethical approval for this study was granted by the Stanford University Institutional Review Board (IRB) (Protocol ID 35921) and Sichuan Univesity Institutional Review Board (IRB) (Protocol ID 2013005–01). All subjects gave written informed consent in accordance with the Declaration of Helsinki.

### Sample selection

Our study was conducted in 22 nationally-designated impoverished counties located in the Qinba mountain region beginning in November 2015. From each of these 22 counties, all townships (the administrative division between counties and villages) were selected to participate in the study. To ensure that we selected a typical rural sample, there were two exceptions to this rule: we excluded the township in each county that housed the county seat, which would typically be more prosperous than the other areas, and we excluded townships that did not have any villages with a population of 800 or more. After imposing these criteria, 115 townships were selected for the study.

We then selected the sample villages and children for this study. To meet the power requirements of a large, interventional study (not reported in this paper), we needed a minimum of five children in each township. With this requirement in mind, we first randomly selected one village from each township to participate. All children in our desired age range of 6–24 months were enrolled in the study. If a village had fewer than five children in our desired age range, we randomly selected an additional village in the same township for inclusion in the study and continued to randomly select additional villages until five children per township had been found. Overall, our study included 1,831 children in 118 villages across 115 townships in rural areas of the Qinba mountain region.

### Data collection

The data presented in this study was collected in four survey waves: November 2015, April 2016, September 2016, and February 2017. In each survey wave, teams of trained enumerators collected two blocks of data. The first block collected socioeconomic information from all households participating in the study. Each toddler’s primary caregiver was identified and administered a detailed survey on parental and household characteristics, including the child’s gender, gestational age, birth weight, mother’s age and education, father’s education, and whether the family received Minimum Living Standard Guarantee Payments from the government (a poverty indicator and a form of government welfare for the lowest income families nationwide). The exact age of each child was obtained from his or her birth certificate. The primary caregiver (typically either the child’s mother or grandmother) was identified in each family as the individual who is most responsible for the child’s care.

In the second survey block, all children were administered two different scales to measure developmental outcomes: the Revised Version of the Bayley Scales of Infant Development and the third version of the Ages and Stages Questionnaire (ASQ-3). As mentioned above, the Bayley Scales is considered the gold standard for identifying developmental delay in young children. The first edition of the Bayley Scales (Bayley-I) was formally adapted to the Chinese language and environment in 1992 [32]. In this study, we used the third edition of the Bayley Scales (Bayley-III). This version of the test was translated into Chinese through a collaboration between Stanford REAP and Shanghai Jiao Tong University in 2015, and then translated back into English. Translators of the Bayley-III gave careful consideration to the cultural context of rural China when translating this test but did not make any significant changes to the content of the test. We also conducted a pilot test of the Bayley-III in rural China before the first survey wave to ensure cultural appropriateness.

The Bayley-III assesses six skillsets of child development: Cognitive, Receptive Language, Expressive Language, Fine Motor, Gross Motor and Social-emotional. The Bayley-III is modified from the original Bayley-I in two ways. The Bayley-III separates the original Mental Developmental Index (MDI) of the earlier versions of the Bayley scales into two distinct scales, Cognitive and Language [33]. The Cognitive Scale assesses play skills, information processing (attention to novelty, habituation, memory, and problem–solving), and counting and number skills. The Language Scale is comprised of Receptive Language and Expressive Language subtests used to assess communication skills, including language and gestures. Additionally, the Psychomotor Developmental Index (PDI) is adjusted into the Motor Scale in the Bayley-III, which is comprised of the Fine Motor subtest and Gross Motor subtests [33,34]. In this study, we use the Cognitive, Receptive Language, Expressive Language, Fine Motor and Gross Motor scales to measure child development.

The test was administered in the home of each child using a set of standardized toys and a detailed scoring sheet. The Bayley-III takes into consideration each child’s age in days, as well as whether he or she was born prematurely. These two factors, combined with the child’s performance on a series of tasks using the standardized toy kits, contribute to the establishment of independent, internationally standardized scores. The primary caregiver was present to hold and comfort the child while trained enumerators conducted the Bayley-III test.

Postgraduates were recruited from different universities to be trained as Bayley-III enumerators. Most of the Bayley-III testers are in early childhood education-related majors, so as to interact well with children ages 5–24 months. All of the enumerators are native Chinese speakers, and most of the enumerators are also from Shaanxi province, so they can understand the local accent. Some of the enumerators had previously been trained to administer the Bayley-III; however, all enumerators (including those who had previously received training) underwent a formal week-long course in administering the Bayley-III. This included 2.5 days of practical training in the field.

The Bayley-III training strictly obeyed the Bayley Administration Manual and contained both theoretical training and practical training. The theoretical training included the administration and interpretation of comprehensive developmental assessments and principles of assessment procedures. The practical training took all enumerators to the field to administer Bayley-III tests to children of similar ages and backgrounds as those in our sample. The practical training tests were supervised by experienced experts to assess whether enumerators were able to establish and maintain rapport, elicit optimum performance, follow standardized administration procedures, understand psychometric statistics, score and interpret the test, and maintain test security. Base on the above aspects, the experts decided whether the enumerators were qualified to administer the Bayley-III test. Those who were deemed unqualified did not enumerate the Bayley-III tests conducted in our survey.

In addition to the Bayley-III test, all children were also given an alternative scale, the ASQ-3, of which we will be able to measure the relevant effectiveness by comparing it to the Bayley-III. The ASQ-3 is an age-specific, child developmental monitoring system designed for caregivers to assess their children's skills, covering all children from 1 month to 5 years of age. In our survey, the ASQ was administered by trained enumerators who interviewed the primary caregiver of each child. During the interview, the primary caregiver remained with the child and interacted with the child. If the caregiver was not sure how to answer a question, they were instructed to interact directly with the child to determine the appropriate answer.

The ASQ consists of 30 simple, straightforward questions regarding five skillsets of childhood development: communication, gross motor, fine motor, problem-solving, and personal-social. The answer to each question is simple, as parents select from three possible responses: “yes,” “sometimes,” or “not yet”. Caregivers are instructed to select "yes" if the child shows a specific behavior; "sometimes" if the particular behavior is occasional or new; or "not yet" if the topic refers to a behavior the child has not yet shown. “Yes” responses add 10 points, “sometimes” responses add 5 points, and “not yet” responses add 0 points. The status of the toddler’s developmental progress can be obtained by comparing the scores of five domains with cutoff scores of the corresponding domain, obtained by empirical study. The ASQ divides children into a series of administration age windows. Each window includes one month on either side of the age interval (e.g., the 6-month age window includes children aged 5 months and 0 days to 6 months and 30 days).

The ASQ was administered by trained enumerators who interviewed the primary caregiver of each child. As with the Bayley-III, the enumerators of the ASQ were postgraduates recruited from different universities; all of the enumerators are native Chinese speakers, and most of the enumerators are from Shaanxi province. Most of the ASQ enumerators are from education or social work-related majors, in order to communicate well with rural caregivers.

All ASQ enumerators received a weeklong formal training in the administration of the ASQ. This training strictly followed the Ages and Stages Questionnaire Manual and included a 2.5 day field training in which enumerators conducted ASQ interviews with the caregivers of children of similar ages and bacgrounds to those in our sample. The field training was supervised by experts who evaluated whether the enumerators used the protocol and language of the ASQ-3 instrument to interview caregivers. The experts then determined whether enumerators were qualified to conduct the ASQ. Only those enumerators who were deemed qualified were permitted to conduct the ASQ in any of the four survey waves.

Enumerators followed all administration requirements for both the ASQ and Bayley-III. Based on these requirements, we used the child’s chronological age to adjust scores This means that if the tested child was born prematurely and under 24 months old, we would adjust for the child's prematurity by subtracting the amount of prematurity from the child's age.

### Statistical analysis

Our statistical analysis is comprised of six parts. First, we analyzed the characteristics of Bayley-III and ASQ-3. Then, we used Cronbach coefficients to interpret the internal consistency of these two instruments for each domain measured. Cronbach coeffecients are calculations of how closely related a set of items are as a group and are considered to be a measure of scale reliability. It is scored between 0 and 1; scores closer to 1 indicate greater internal consistency, meaning that we can have greater trust in this scale.

Second, we measure delays in each of the two instruments. In the instruction of Bayley-III, the cutoff points are (1) lower than mean by 1 SD and (2) lower than mean by 2 SD. In the instruction of ASQ-3, the cutoff points is lower than mean by 1.5–2.0 SD. In our study both the Bayley-III and the ASQ-3 use two criteria to define delay: (1) lower than the mean by 1 SD and (2) lower than the mean by 2 SD. Although the instruction of the ASQ-3 did not set the score lower than mean by 1 SD as a cut-off score, we still use it due to following reasons. First, 1 SD is a critical cutoff point for ASQ-3 since children whose scores below 1.0 SD by mean were recommended to get a diagnostic test [35]. Second, the 1 SD cut-off score corresponds to the Bayley-III cutoff scores, as Bayley-III uses 1 SD and 2 SD as cutoff points. Finally, although 1.5 SD, in addition to 2 SD, was recommended by the ASQ instructions, the 1.5 SD cutoff is not commonly used in the literature, while 1SD or 2 SD are generally more commonly used [18,25,26]. Based on the reasons listed above, we chose to use the 1 SD cut-off rather than the 1.5 SD cutoff. Since Bayley-III and ASQ-3 criteria specific to China are unavailable, we used the criteria for defining delays (Bayley-III) and risk of delays (ASQ) in American children.

Third, we measured the validity of the domains assessed by the ASQ-3. In order to examine the validity of every individual skillset of ASQ-3, we categorize the skillsets of both scales into three domains: cognitive development, language development and motor development. In our study, a domain refers to a general development ability measured by both scales. This is based on the principle that each test assesses the same or similar skillsets. Therefore, we categorize both the communication skillset in ASQ-3 and the language skillset in the Bayley-III into the language domain; we categorize gross motor and fine motor skillsets from both tests into the motor domain; and we categorize the problem-solving skillset in ASQ-3 and the cognitive skillset in the Bayley-III in the cognitive domain. We then calculated the proportion of children with delays in each specific developmental domain (e.g. cognitive development)

We then measured the sensitivity and specificity of the ASQ as compared to Bayley-III. Sensitivity measures the proportion of children who were identified as at risk for developmental delay according to the ASQ-3 and were identified as such by the Bayley-III. Specificity measures the proportion of children who were identified as normal by the ASQ-3 and did not show a delay on the Bayley-III. We measure sensitivity and specificity by examining what we refer to as true positives, false positives, true negatives and false negatives. True positives refer to the percentage of children identified by the Bayley-III as having a delay who were also identified by the ASQ 3 as being “at risk.” False positives refer to the percentage of children identified by the Bayley-III as normal but who were identified by the ASQ 3 as being “at risk.” True negatives refer to the percentage of children identified by the Bayley-III as normal who were also identified by the ASQ 3 as normal. Finally, false negatives refers to the percentage of children identified by the Bayley-III as having a delay but who were identified by the ASQ-3 as being normal.

In the literature to date, there is no agreed-upon cut-off for the percentage of true positives and true negatives that can be seen as sufficient to determine the accuracy of detecting developmental delays. In fact, few studies have set cut-off scores to determine the validity of psychometric tests. However, Steenis et al., (2015) used 70% as the cut-off of sensitivity and specificity to measure the validity of scales such as the ASQ-3 [18]. Following their methods, in this study, we also set 70% as the cut-off for determining validity, which means ASQ-3 can be seen as a good screening instrument if the values for sensitivity and specificity are 70% or higher.

In addition, we calculated the Pearson correlation between Bayley-III and ASQ-3 for each of the domains. Regarding the effect size of our Pearson correlation, estimates r (the strength of the correlation), we will use the general guidelines: if the absolute value of Pearson correlation is between 0.1 & 0.3, then we have small correlation, if the absolute value of Pearson correlation is between 0.3 & 0.5, then we have medium/moderate correlation, and if this value is above 0.5, then we have large/strong correlation. P-values of the correlations were calculated by bootstrapping methods, with 1,000 replications and clustering by socioeconomic factors, which was completed using principal component analysis scores (as known as PCA Scores) divided into three strata by the cut-off of 25% and 75% quantiles.

Finally, we calculated the correlations between the scores of these two tests and a set of variables shown to be related to child development. These included maternal education, family economic status, the variety of play activities, and play materials. Because all of these variables are recognized to be factors related to child development, this is one way to determine the similarity of the two tests in terms of how they identify developmental delays and can help us better understand the results of our other analyses.

All statistical analysis was performed using Stata 14.1 statistical software. When calculating the correlations of these two tests, we divided the samples into age cohorts of 5–12 months, 13–18 months, and 19–24 months. All correlations were Pearson correlations, and p-values below 0.05 were considered significant.

## Results

The basic characteristics of our sample are displayed in Table 1. As can be seen in the table, of the 1,831 children in this study, slightly over half (52%) were male–a ratio that reflects the overall gender imbalance in China [36]. Around 5% of the children in the study were born prematurely and 11% of households reported receiving Minimum Living Standard Guarantee Payments. For 25.1% of the children in our sample, the grandmother was identified as the primary caregiver; for the other 74.9% of children, the mother was most often the primary caregiver. Educational attainment was low: overall, the average maternal years of education was 9.2, and the average paternal years of education was 9.5.

**Table 1:** Characteristics of children in the study sample.

Characteristics	
1. Toddler's age, %	
5–12 months	38.7
13–18 months	34.4
19–24 months	26.9
2. Receives welfare subsidy, %	11.1
3. Girls, %	48
4. Premature (gestional age <37 weeks), %	4.7
5. Birth weight, g, mean (SD)	3260 (462)
6. Stunted (z-score height-for-age < -2SD)	3.28
7. Mother's age, y, mean (SD)	27.9 (4.9)
8. Mother's education, y, mean (SD)	9.2 (2.7)
9. Father's education, y, mean (SD)	9.5 (2.6)
10.Caregiver is grandmother, %	25.1

### Early childhood development in rural Shaanxi province: Results of the Bayley-III

Bayley-III and ASQ-3 measures were available for all 1,831 children. The mean scores (SD) of developmental outcomes of our sample children are presented in Table 2. Mean composite scores of the Bayley-III were within the normal range, although standard deviations (SD) were low, further justifying the pertinence of the internal standardization. Mean ASQ scores in our data are less than the normal mean ASQ scores reported in the ASQ-3 Guide. This means that the development of the children in our sample is, overall, behind that of the US normal sample.

**Table 2:** The summary statistics of ASQ-3 and Bayley-III.

	Mean (SD)	Median	Min	Max
1. Bayley-III raw scores				
Cognitive	44.1 (11.3)	45	7	88
Receptive Language	16.2 (6.1)	15	3	74
Expressive Language	16.5 (7.5)	16	0	37
Fine Motor	30.6 (7.3)	32	1.9	89
Gross Motor	42.7 (12.9)	46	10	153
2. Bayley-III scaled scores				
Cognitive	9.2 (2.5)	9	1	16
Receptive Language	8.7 (2.8)	9	1	18
Expressive Language	8.7 (2.4)	9	1	16
Fine Motor	10.1 (2.8)	10	1	18
Gross Motor	8.9 (3.6)	9	1	20
3. Bayley-III composite scores				
Cognitive	96.0 (12.6)	95	55	135
Language	92.3 (13.8)	91	50	135
Motor	97.3 (16.7)	97	46	175
4. ASQ-3				
Problem Solving	43.2 (13.9)	45	0	60
Communication	39.2 (14.0)	40	0	60
Fine Motor	42.3 (13.7)	45	0	60
Gross Motor	42.2 (15.6)	45	0	60
Personal-Social	40.3 (13.8)	40	0	60

### Comparison of the ASQ-3 and Bayley-III

Before presenting our main results, we first report the psychometric characteristics of the ASQ-3, including sensitivity and specificity (Table 3). Overall, we find that the sensitivity and specificity of the ASQ-3 are 76.52% and 40.97%, respectively, by the 1SD cutoff as well as 66.54% and 62.79%, respectively, by the 2SD cutoff. Moreover, we find that more than two third of sensitivities and specificities are below the 70% of cut-off scores.

**Table 3:** Pass/fail agreement between the ASQ-3 and Bayley-III.

Cut-off	Age cohorts	Domain	Risk of delay in ASQ-3 (%)	Delay Bayley-III (%)	True positives (%)	False positives (%)	True negatives (%)	False negatives (%)	Sensitivity	Specificity
1SD	5–12 months	cognitive	41.6	37.7	19.5	22.1	40.2	18.2	51.7%	64.5%
		language	42.5	60.5	28.5	14.0	25.5	32.0	47.1%	64.6%
		motor	74.5	67.4	54.9	19.6	13.0	12.6	81.3%	39.9%
	13–18 months	cognitive	26.7	29.0	11.1	15.6	55.4	17.9	38.3%	78.0%
		language	36.8	49.5	24.1	12.7	37.8	25.4	48.7%	74.9%
		motor	52.5	20.0	16.0	36.5	43.5	4.0	80.0%	54.4%
	19–24 months	cognitive	17.1	45.5	8.5	8.5	45.9	37.0	18.7%	84.4%
		language	17.7	42.9	12.0	5.7	51.4	30.9	28.0%	90.0%
		motor	33.9	10.6	5.3	28.7	60.8	5.3	50.0%	67.9%
2SD	5–12 months	cognitive	21.4	8.6	3.2	18.2	73.2	5.4	37.2%	80.1%
		language	15.9	19.2	3.4	12.6	68.3	15.8	17.7%	84.4%
		motor	47.8	34.3	21.6	26.2	39.5	12.7	63.0%	60.1%
	13–18 months	cognitive	14.4	5.1	1.9	12.5	82.4	3.2	37.3%	86.8%
		language	6.7	14.6	3.2	3.5	81.9	11.4	21.9%	95.9%
		motor	27.5	5.2	3.5	24.0	70.8	1.7	67.3%	74.7%
	19–24 months	cognitive	6.7	4.3	0.8	5.9	89.8	3.5	18.6%	93.8%
		language	7.5	7.7	2.2	5.3	87.0	5.5	28.6%	94.3%
		motor	17.5	2.4	1.0	16.5	81.1	1.4	41.7%	83.1%

Note: (1)True positives refer to when both the ASQ-3 and Bayley-III test indicate delay; False positives refers to when the ASQ-3 indicates a risk of delay but the Bayley-III test indicates development is in the normal range; True negatives refers to when both the ASQ-3 and the Bayley-III test indicate that development in the normal range; False negatives refers to when the ASQ-3 indicates that development is in the normal range but the Bayley-III test indicates a risk of delay. (2) The cut-off scores of the first column are both for Bayley-III or ASQ 3 because we compared these two tools by using the same cut-off score (1 SD cut-off and 2 SD cut-off); (3) The delay of any part of the fine motor or the gross motor will be considered delay in the "motor" domain in the ASQ 3 part.

We also have several findings regarding specific trends. First, when the cut-off score is specified as less than 1SD, ASQ-3 has a higher sensitivity (the ability to correctly identify developmental delays) and lower specificity (the ability to correctly identify normal-range development) in comparison to the 2SD cut-off score. Second, the motor domain has a higher sensitivity than the cognitive and language domains, and the language domain has a higher specificity than the cognitive and motor domains, regardless of the cut-off score. Third, the specificity (but not sensitivity) increases with the age of children in our sample.

Next, we looked at the correlation between the ASQ-3 and Bayley-III scores (Table 4), finding that in general the concurrence of the two tests increased with the age of the children. The ASQ-3 problem solving, communication, gross motor, and personal-social scales had similarly weak but significant correlations with the corresponding Bayley-III scales at 5–12 months. In this age group, however, the ASQ-3 fine motor scale did not significantly correlate with the Bayley-III scale. Concurrence was higher for the 13–18 month group across all scales, and in this group the correlation of the fine motor scales was significant. In the over 18 month age group, the correlations remained significant for all domains, although the coefficient decreased for the problem solving (cognitive) and gross motor scales.

**Table 4:** Correlations among Bayley-III Scales and between scales in the Bayley-III and the ASQ-3 by age group.

	Bayley III, 5–12 months						Bayley III, 13–18 months					Bayley III, 19–24 months				
	Cognitive	Receptive Language		Expressive Language	Fine Motor	Gross Motor	Cognitive	Receptive Language	Expressive Language	Fine Motor	Gross Motor	Cognitive	Receptive Language	Expressive Language	Fine Motor	Gross Motor
**Bayley-III**	n = 709						n = 630					n = 492				
Cognitive	1						1					1				
Receptive Language	0.616***		1				0.672***	1				0.49***	1			
Expressive Language	0.623***		0.686***	1			0.597***	0.742***	1			0.474***	0.612***	1		
Fine Motor	0.778***		0.606***	0.65***	1		0.514***	0.461***	0.403***	1		0.53***	0.435***	0.505***	1	
Gross Motor	0.743***		0.605***	0.658***	0.805***	1	0.518***	0.476***	0.513***	0.392***	1	0.426***	0.422***	0.461***	0.519***	1
**ASQ-3**	n = 709						n = 630					n = 492				
Problem Solving	0.173***		0.132***	0.108**	0.226***	0.201***	0.264***	0.151***	0.141***	0.156*	0.22***	0.119*	0.153***	0.154***	0.153***	0.136**
Communication	0.107**		0.152***	0.157***	0.091*	0.126***	0.173***	0.251***	0.332***	0.122**	0.191***	0.268***	0.338***	0.491***	0.309***	0.273***
Fine Motor	0.061		0.02	0.007	**0.07**	0.059.	0.324***	0.264***	0.255***	0.222***	0.262***	0.17***	0.174***	0.163***	0.25***	0.143**
Gross Motor	0.178***		0.086*	0.077*	0.179***	0.341***	0.345***	0.263***	0.248***	0.274***	0.493***	0.212***	0.164***	0.17***	0.146**	0.317***
Personal-Social	0.204***		0.099**	0.085*	0.238***	0.243***	0.276***	0.240***	0.253***	0.146*	0.198***	0.143**	0.158***	0.156***	0.148**	0.103*

Note: Pearson correlations on internally standardised scores; Standard Errors (SE) computed using bootstrap stratifying by age category

* p<0.05

** p<0.01

*** p<0.001.

From comparisons between different subgroups, we also found that the identity of the primary caregiver affects how well the ASQ-3 corresponds with the Bayley-III (Table 5 and Table 6). When the grandmother is the primary caregiver, we found that the ASQ-3 scales consistently had weaker correlations with the Bayley-III than when the mother is the primary caregiver. For the 5–12 months age groups, the ASQ-3 problem solving, gross motor, and personal-social scale scores do not significantly predict the Bayley-III scale scores when the grandmother is the primary caregiver. However, when the primary caregiver is the mother, the ASQ-3 problem solving, gross motor, and personal-social scales are significantly correlated with the corresponding Bayley-III scales at 5–12 months.

**Table 5:** Correlations among Bayley-III scales and between scales in the Bayley-III and the ASQ-3 by age group (Grandmother is the primary caregiver).

	Bayley III, 5–12 months					Bayley III, 13–18 months					Bayley III, 19–24 months				
	Cognitive	Receptive Language	Expressive Language	Fine Motor	Gross Motor	Cognitive	Receptive Language	Expressive Language	Fine Motor	Gross Motor	Cognitive	Receptive Language	Expressive Language	Fine Motor	Gross Motor
**Bayley-III**	n = 115					n = 181					n = 163				
Cognitive	1					1					1				
Receptive Language	0.704***	1				0.722***	1				0.495***	1			
Expressive Language	0.614***	0.72***	1			0.599***	0.779***	1			0.460***	0.548***	1		
Fine Motor	0.842***	0.661***	0.613***	1		0.364	0.379*	0.281	1		0.589***	0.428***	0.509***	1	
Gross Motor	0.761***	0.575***	0.543***	0.812***	1	0.641***	0.585***	0.614***	0.323*	1	0.511***	0.443***	0.502***	0.529***	1
**ASQ-3**	n = 115					n = 181					n = 163				
Problem Solving	**0.156**	0.166	0.041	0.101	0.153	0.166*	0.081.	0.047	-0.039	0.127	0.168*	0.139	0.128	0.125	0.152*
Communication	0.229*	0.214*	0.185*	0.206*	0.198*	0.082	0.246***	0.326***	0.046	0.111	0.312***	0.279***	0.522***	0.319***	0.330***
Fine Motor	0.030	0	-0.039	**0.011**	0.080	0.246***	0.234***	0.219***	**0.079**	0.275***	0.165*	0.158*	0.149*	0.196**	0.143
Gross Motor	0.176*	-0.010	0.020	0.139	0.359***	0.316***	0.248***	0.225***	0.168	0.568***	0.238***	0.201***	0.227**	0.245***	0.372***
Personal-Social	0.212*	0.067	0.022	0.145	0.196*	0.318***	0.269***	0.258***	0.073	0.24***	0.168*	0.206**	0.140	0.114	0.196*

Note: Pearson correlations on internally standardised scores; Standard Errors (SE) computed using bootstrap stratifying by age category

* p<0.05

** p<0.01

*** p<0.001.

**Table 6:** Correlations among Bayley-III scales and between scales in the Bayley-III and the ASQ-3 by age group (Mom is the primary caregiver).

	Bayley III, 5–12 months					Bayley III, 13–18 months					Bayley III, 19–24 months				
	Cognitive	Receptive Language	Expressive Language	Fine Motor	Gross Motor	Cognitive	Receptive Language	Expressive Language	Fine Motor	Gross Motor	Cognitive	Receptive Language	Expressive Language	Fine Motor	Gross Motor
**Bayley-III**	n = 567					n = 415					n = 293				
Cognitive	1					1					1				
Receptive Language	0.599***	1				0.658***	1				0.492***	1			
Expressive Language	0.619***	0.68***	1			0.616***	0.724***	1			0.466***	0.669***	1		
Fine Motor	0.759***	0.599***	0.658***	1		0.646***	0.541***	0.514***	1		0.496***	0.433***	0.498***	1	
Gross Motor	0.736***	0.616***	0.683***	0.807***	1	0.484***	0.442***	0.484***	0.458***	1	0.381***	0.426***	0.452***	0.519***	1
**ASQ-3**	567					415					293				
Problem Solving	0.171***	0.114**	0.117**	0.253***	0.209***	0.306***	0.193***	0.183***	0.308***	0.264***	**0.105**	0.168**	0.187***	0.157**	0.161*
Communication	0.079	0.139***	0.151***	0.073	0.114**	0.232***	0.260***	0.335***	0.215***	0.228***	0.243***	0.37***	0.484***	0.300***	0.248***
Fine Motor	0.054	0.010	0.015	**0.072**	0.044	0.368***	0.288***	0.285***	0.344***	0.271***	0.197**	0.171**	0.175**	0.272***	0.162**
Gross Motor	0.165***	0.104*	0.086*	0.182***	0.324***	0.353***	0.261***	0.243***	0.364***	0.468***	0.228***	0.156**	0.164**	0.095	0.303***
Personal-Social	0.191***	0.102*	0.089*	0.247***	0.251***	0.290***	0.228***	0.243***	0.233***	0.200***	0.160**	0.141*	0.181**	0.157*	0.089

Note: Pearson correlations on internally standardized scores; Standard Errors (SE) computed using bootstrap stratifying by age category

* p<0.05

** p<0.01

*** p<0.001.

### Correlations with other variables

Table 7 (Table B in S1 Appendix) presents correlations between Bayley-III/ASQ-3 and factors related to child development. In the combined age groups, all of the Bayley-III scales were significantly correlated with play activities and play materials factors and cognitive, receptive language and expressive index of the Bayley-III showed significant correlations with maternal education when we controlled other charactieristics (such as, gender, prematurity, assets). All scales except for fine motor were also significantly correlated with the wealth index. Regarding the ASQ-3, all scales except for communication were not significantly correlated with maternal education, but all scales were significantly correlated with play activities, and play materials. We also found all scales showed a significant correlation with the wealth index when we controlled other charactieristics. When we use Bayley scaled scores, we see the same significant results (Table B in S1 Appendix).

**Table 7:** Correlations of the Bayley-III and the ASQ-3 with maternal education, household wealth, play activities and play materials in the home, all ages combined activities and play materials in the home, all ages combined.

	Maternal Education^a^	Wealth Index^b^	Play Activities^b^	Play Materials^b^
**Bayley-III**				
Cognitive	0.040***	0.023***	0.014	0.037***
Receptive Language	0.064***	0.053***	0.039***	0.062***
Expressive Language	0.051***	0.046***	0.031**	0.043***
Fine Motor	0.040*	0.023**	0.024*	0.061***
Gross Motor	0.006	0.020**	0.018**	0.037***
**ASQ-3**				
Problem Solving	1.142	1.705***	0.163***	0.251***
Communication	2.790**	1.590***	0.215***	0.236***
Fine Motor	1.635	1.883***	0.176***	0.228***
Gross Motor	-2.302	1.558***	0.147***	0.231***
Personal-Social	0.687	1.501***	0.161***	0.225***

^a^ shows coefficients from OLS regressions additionally controlling for child’s age, child’s gender, whether the child was premature, caregiver type, age of mother, educational level of father, asset index, whether the household receives government welfare.

^b^ shows coefficients from OLS regressions additionally controlling for child’s age, child’s gender, whether the child was premature, caregiver type, age of mother, educational level of father, maternal educational level, whether the household receives government welfare.

* p<0.05

** p<0.01

*** p<0.001.

To check these results, we used the composite Bayley-III scores and the ASQ-3 scores computed using the original six-item questionnaires; we also divided the sample by different age groups. The results were not significantly different.

## Discussion

The goal of this study has been to examine whether the ASQ-3 is an accurate screening measure for children at risk of developmental delays in rural areas of China. We judged this by comparing results from the ASQ-3 with child performance on the Bayley-III, a large-scale diagnostic test considered the “gold standard” of early childhood development tests. To meet this goal, we collected unique data on 1,831 caregiver-child dyads in rural Shaanxi province. We then assessed the levels of development among the sample children using both ASQ-3 and Bayley-III scales.

We found that the ASQ-3 was significantly though weakly correlated with the Bayley-III across all indices and that the strength of this correlation increased with age. Scores from the problem solving (or cognition), communication (or language), motor, and personal social (or social-emotional) domains of the ASQ-3 and Bayley-III were weakly though significantly correlated across all age groups. The fine motor scales of the ASQ-3 and Bayley-III were not significantly correlated in the 5–12 month age group and then showed weak but significant validity in the older age groups. In addition, we found that, across all scales, the concurrence generally had a direct relationship with children’s age. These results reflect those of several studies mentioned earlier which found that the validity of the ASQ-3 improved (indicated by stronger correlations with the Bayley-III) as the age of sample children increased [17,18,27].

When we compared the sensitivity and specificity of the ASQ-3 and Bayley-III-III, we found sensitivity values ranging from 17.7% to 81.3%. Most of the sensitivity values (16 out of 18) were below the 70% of cut-off, which indicates that most of the children who were identified as at risk for developmental delay according to the ASQ-3 were not identified as such by the Bayley-III. Specificity values also ranged widely, from 39.9% to 95.9%. Of these, although over half of the specificity values were greater than 70%, just under half (7 out of 18) were lower than the 70% cut-off score, which indicates that many children who were not identified as at risk for delay by the ASQ-3 did show a delay on the Bayley-III. These results are similar to Steenis et al. [18], which also found wide-ranging sensitivity (7% to 77%) and specificity (53% to 99%) values. In addition, we discovered that the specificity values were generally higher for the older age-cohort than for the younger age-cohort children. These results differ somewhat from other studies [18,27,37], which found that not only the sensitivity but also the specificity increased with toddler age. The differences between our findings may be due to differences between toddler age, sampling areas, or the criteria used to define delay.

These results indicate that, overall, the ASQ-3 may be more accurate when used to test older-aged children, though there is still a statistically significant correlation between the two tests in the earliest age group of 5–12 months. In this way, these results reflect the conclusions of previous studies that also indicate the ASQ-3 shows the best potential as a screener of older children [18,27]. However, these findings contrast with the findings of some previous researchers, in that in the youngest age groups the correlations between the two tests were significant overall, albeit weak. Rubio-Codina et al., for example, found that when the children are under 31 months, ASQ-3 was not significantly correlated with the Bayley-III across most indices [17]. This is perhaps due to variations in sampling.Ultimately, however, our findings lead us to agree with the conclusions of Rubio-Codina et al. (2016), that the ASQ-3 may not be an accurate screening tool for identifying developmental delays in younger children.

This poor validity is concerning, as the ASQ-3 is becoming more and more commonly used in large survey studies [38,39]. According to our findings, this entails that many children who are identified as developmentally delayed by the Bayley-III are not identified as such by the ASQ-3 (indicating poor sensitivity). This discrepancy might at least partially be explained by a mismatch between caregiver knowledge and toddler developmental level: as some caregivers might not spend much time with their children and/or be aware of the toddler’s activities or abilities, they might not be able to accurately answer the questions in such surveys.

When comparing different subgroups, we found that administering the ASQ-3 to mothers may result in a higher correlation with the Bayley-III than when the test was administered to grandmothers (Tables 5 and 6). This finding is bolstered by a recent study conducted in rural China that showed that grandmothers who are primary caregivers are less engaged with children when compared to mothers who are primary caregivers [28]. This phenomenon does not seem to appear in studies that have been conducted in places outside of China [17–19,25–27,40–42], however, suggesting that this trend is unique to rural China due to the increasing number of children left behind by their parents to stay with surrogate caregivers (most often grandmothers) as rural-urban migration continues to proliferate [43].

Finally, regarding the correlations of the ASQ and Bayley-III with other variables, we find that both Bayley-III and ASQ-3 domains were all significantly correlated with play activities and play materials. However, whereas all domains of Bayley-III is significantly correlated with maternal education, all domains of the ASQ except for communication were not significantly correlated with maternal education. The correlation between maternal education and child development has been well established both theoretically and empirically [44–46]. That we found no significant correlations between the ASQ-3 and maternal education is not only inconsistent with the Bayley-III; it is also inconsistent with the early childhood development literature overall. This means that ASQ may not measure child development as comprehensively or as accurately as Bayley-III.

Taken together, these findings indicate that that the ASQ may not be able to measure the development of children with great consistency or accuracy as compared to the Bayley-III. One possible reason for this may be due to the caregiver reporting structure of the ASQ. Primary caregivers may struggle to understand how their children are developing, leading to inaccurate responses. This may be in part due to lack of interactive parenting practices, leading parents to know less about their children’s abilities. That mothers returned better results than grandmothers supports this hypothesis. Past research has found that grandmothers are less likely to engage in interactive parenting than mothers [47], meaning they are less attuned to the development of the child. If this is the case, then one of the only ways to improve the sensitivity and specificity of the ASQ would be to increase interactive parenting among parents of young children in rural China. Future research should also use qualitative methods to understand how the ASQ can be better adapted to the context of families in rural China, which may help caregivers to give more accurate answers.

This study shows that the ASQ-3 may not be a viable supplement or replacement for the Bayley-III to test children, though it has certain advantages when compared with the Bayley-III and other tests. As compared with diagnostic ECD tests like the Bayley-III, we found that ASQ-3 has been shown to be both cost-effective and easily administered [48]. The ASQ-3 test is still one of the most feasible to administer because it is short, inexpensive, and requires little training. In addition, this is also one of the only multi-dimensional developmental tests that can be used in surveys, and in particular large-scale surveys. However, our results demonstrate that large-scale multi-dimensional diagnostic tests, such as the Bayley-III, are still the most valid and accurate, as argued by a number of other scholars [17,26,49].

### Study limitations and strengths

We acknowledge a few limitations to this study as well as several of its strengths. One limitation of the study is that we do not use longitudinal follow-up data to test the correlation between ASQ-3 and Bayley-III over time. Our research team is currently preparing one long-term survey to examine the concurrent validity annually. A second limitation is that we only examined the development of children up to 24 months, so we cannot judge the validity of the ASQ-3 among children older than two years. A third limitation is that the version of the Bayley-III and ASQ tests we use have not yet been administered to a healthy reference population in China. As such, we rely on reference populations from the United States for both tests [34,50,51]. Future studies should create norms for Chinese reference populations and reassess the concurrent validity of the ASQ-3 and the Bayley-III to confirm the findings of this study. Fourth, although the order of the Bayley-III test and the ASQ-3 test were random, there was a small share of primary caregivers who were administered the ASQ-3 after the Bayley-III was completed. In these cases, it is possible that test fatigue affected the quality of their responses to the ASQ-3. Finally, although we conduct several statistical tests to provide evidence on the validity of the ASQ-3, this may not be a sufficient accumulation of evidence to determine the validity of the ASQ-3 with confidence. Future research should consider how to test the correlation between the ASQ-3 and Bayley-III using longitudinal and randomized study data, so as to provide further evidence of the validity of ASQ-3.

The strengths of this study include its large sample size (1,831 children) and the fact that this is the first time that the validity of the ASQ-3 has ever been tested against the Bayley-III in China.

## Conclusion

Overall, our findings suggest that there is a weak but significant relationship between ASQ-3 and Bayley-III. Specifically, ASQ-3 generally performed poorly on assessing the development of children under 13 months old. We also found that the sensitivity and specificity are widely distributed (17.7–95.9). Our study suggests that the ASQ-3 is better at screening older children than younger children for developmental delays. The ASQ-3 correctly identified the large majority of older children aged 19–24 months old who do not have a developmental delay according to the Bayley-III (indicating high specificity for older children), but it still was unable to correctly identify many of those children who did have developmental delays according to the Bayley-III, regardless of age (indicating low sensitivity overall). Finally, we found that the accuracy of the ASQ-3 depended on the identity of the caregiver in question. We found that the ASQ-3 corresponded more closely to the Bayley-III when the mother (as opposed to the toddler’s grandmother) was identified as the primary caregiver.

All in all, measuring the development of young children is a challenging task. As discussed in the previous section, while our results indicate that the ASQ-3 cannot be used as a replacement for the Bayley-III, there are certain contexts in which the ASQ-3 could potentially at least be used in conjunction with the Bayley-III, depending on the domain in question and the age of the children.

To avoid the long-term negative consequences that poor early childhood ourcomes have on human capital development, China’s government should be aware of the state of its nation’s children and take steps to improve the early development of children in rural areas. One government agency that could take such an initiative is the Population and Family Planning Commission (PFPC), which has relatively easy access to villages in China. Following the end of China’s One-Child policy, the PFPC is looking for a new institutional mission, and it has turned its attention to early childhood development [52]. The PFPC already has institutional reach and experience in conducting village outreach and survey tests using different tools by domain and child age. By implementing surveys of this nature, China can better help a large portion of its nation’s youth develop to their full potential.

## Supporting information

S1 Appendix Table A. Characteristics of the Bayley-III and the ASQ-3
Table B. Correlations of the Bayley-III and the ASQ with Maternal Education, Household Wealth, Play Activities and Play Materials in the Home, All Ages Combines (Using Bayley scaled scores)
Table C. Pass/fail agreement between the ASQ and Bayley (Mom is the primary caregiver)
Table D. Pass/fail agreement between the ASQ and Bayley (Grandmother is the primary caregiver).
(DOCX)

## References

[1] Almond D, Currie J. Human capital development before age five. Vol. 4b, Handbook of Labor Economics. 2011. 1315–1486 p.

[2] Knudsen EI, Heckman JJ, Cameron JL, Shonkoff JP. Economic, neurobiological, and behavior perspectives on building America’s future workforce. PNAS. 2006;103(27):10155–62. 10.1073/pnas.0600888103 16801553 PMC1502427

[3] Cunha F, Heckman J, Schennach S. Estimating the technology of cognitive and noncognitive skill formation. Econometrica. 2010;78(3):883–931. 10.3982/ECTA6551 20563300 PMC2885826

[4] Kautz T, Lafontaine P, Raval D, Shaikh A, Smith J, Stigler S. Analyzing social experiments as implemented: A reexamination of the evidence from the HighScope Perry Preschool Program. Quant Econom. 2010;1(1):1–46. 23255883 10.3982/qe8 PMC3524308

[5] Attanasio O, Cattan S, Fitzsimons E, Meghir C, Rubio-Codina M. Estimating the production function for human capital: Results from a randomized control trial in Colombia. Natl Bur Econ Res. 2015;

[6] Manning M., Patterson J. Lifetime Effects: The High/Scope Perry preschool study through age 40. Child Educ. 2006;83(2):121 10.1007/s11060-006-9265-3

[7] Walker SP, Wachs TD, Grantham-Mcgregor S, Black MM, Nelson CA, Huffman SL, et al Inequality in early childhood: Risk and protective factors for early child development. Lancet. 2011;378(9799):1325–38. 10.1016/S0140-6736(11)60555-2 21944375

[8] Gertler P, Heckman J, Pinto R, Zanolini A, Vermeersch C, Walker S, et al Labor market returns to an early childhood stimulation intervention in Jamaica. Science (80-). 2014;344(6187):998–1001. 24876490 10.1126/science.1251178 PMC4574862

[9] Black MM, Walker SP, Fernald LCH, Andersen CT, DiGirolamo AM, Lu C, et al Early childhood development coming of age: science through the life course. Lancet. 2017;389(10064):77–90. 10.1016/S0140-6736(16)31389-7 27717614 PMC5884058

[10] Grantham-McGregor S, Cheung YB, Cueto S, Glewwe P, Richter L, Strupp B. Developmental potential in the first 5 years for children in developing countries. Lancet. 2007;369(9555):60–70. 10.1016/S0140-6736(07)60032-4 17208643 PMC2270351

[11] Sylvia S, Warrinnier N, Renfu L, Yue A, Attanasio O, Medina A, et al From quantity to quality: Delivering a home-based parenting intervention through China’s family planning cadres. LICOS Cent Institutions Econ Perform. 2018;

[12] Landry SH, Smith KE, Swank PR. Responsive parenting: Establishing early foundations for social, communication, and independent problem-solving skills. Dev Psychol. 2006;42(4):627–42. 10.1037/0012-1649.42.4.627 16802896

[13] Parker FL, Boak AY, Griffin KW, Ripple C, Peay L. Parent-Child Relationship, Home Learning Environment, and School Readiness. School Psych Rev. 1999;28(3):413–25.

[14] Heckman JJ. Policies to foster human capital. Res Econ. 2000;54(1):3–56.

[15] Bayley N. Bayley scales of infant development: Manual. Psychological Corporation; 1993.

[16] Bayley N. Bayley Scales of Infant Development 3rd Edition Psychological Corporation; 2006.

[17] Rubio-Codina M, Araujo MC, Attanasio O, Muñoz P, Grantham-McGregor S. Concurrent validity and feasibility of short tests currently used to measure early childhood development in large scale studies. PLoS One. 2016;11(8):1–17. 10.1371/journal.pone.0160962 PMC4993374 27548634

[18] Steenis LJP, Verhoeven M, Hessen DJ, van Baar AL. Parental and professional assessment of early child development: The ASQ-3 and the Bayley-III-NL. Early Hum Dev. 2015;91(3):217–25. 10.1016/j.earlhumdev.2015.01.008 25703316

[19] Limbos MM, Joyce DP. Comparison of the ASQ and PEDS in screening for developmental delay in children presenting for primary care. J Dev Behav Pediatr. 2011;32(7):499–511. 10.1097/DBP.0b013e31822552e9 21760526

[20] Borsboom D, Mellenbergh GJ, Van Heerden J. 63–03 The concept of validity. Psychol Rev. 2004;111(4):1061–71. 10.1037/0033-295X.111.4.1061 15482073

[21] Shepard LA. Chapter 9: Evaluating Test Validity. Vol. 19, Review of Research in Education. 1993. 405–450 p.

[22] Goodwin LD, Leech NL. The meaning of validity in the new standards for educational and psychological testing: Implications for measurement courses. Meas Eval Couns Dev. 2003;36(3):181–91.

[23] Bian X, Yao G, Squires J, Hoselton R, Chen CI, Murphy K, et al Translation and use of parent-completed developmental screening test in Shanghai. J Early Child Res. 2012;10(2):162–75.

[24] Schonhaut L, Armijo I, Schonstedt M, Alvarez J, Cordero M. 61–09 Validity of the Ages and Stages Questionnaires in Term and Preterm Infants. Pediatrics. 2013;131(5):e1468–74. 10.1542/peds.2012-3313 23629619

[25] Gollenberg AL, Lynch CD, Jackson LW, McGuinness BM, Msall ME. Concurrent validity of the parent-completed Ages and Stages Questionnaires, 2nd Ed. with the Bayley Scales of Infant Development II in a low-risk sample. Child Care Health Dev. 2010;36(4):485–90. 10.1111/j.1365-2214.2009.01041.x 20030657

[26] Woodward BJ, Papile LA, Lowe JR, Laadt VL, Shaffer ML, Montman R, et al Use of the ages and stages questionnaire and bayley scales of infant development-II in neurodevelopmental follow-up of extremely low birth weight infants. J Perinatol. 2011;31(10):641–6. 10.1038/jp.2011.1 21311498 PMC3139816

[27] Schonhaut L, Armijo I, Schonstedt M, Alvarez J, Cordero M. Validity of the Ages and Stages Questionnaires in term and preterm infants. Pediatrics. 2013;131(5):e1468–74. 10.1542/peds.2012-3313 23629619

[28] Yue A, Shi Y, Luo R, Chen J, Garth J, Zhang J, et al China’s invisible crisis: Cognitive delays among rural toddlers and the absence of modern parenting. China J. 2017;78(78):50–80.

[29] Luo R, Jia F, Yue A, Zhang L, Lyu Q, Shi Y, et al Passive parenting and its association with early child development. Early Child Dev Care. 2017;1–15.

[30] Bornstein MH, Tal J, Catherine Tamis-LeMonda. Parenting in cross-cultural perspective: The United States, France, and Japan. 1991.

[31] Duan C, Lv L, Guo J, Wang Z. Survival and development of left-behind children in rural China: Based on the analysis of sixth census data. Popul J. 2013;35(3):37–49.

[32] Yi S, Luo X, Yang Z, Wan G. The revising of Bayley scales of infant development (BSID) in China. Chinese J Clin Psychol. 1993;(2):71–5.

[33] Bos AF. Bayley-II or Bayley-III: What do the scores tell us? Dev Med Child Neurol. 2013;55(11):978–9. 10.1111/dmcn.12234 23930736

[34] Lowe JR, Erickson SJ, Schrader R, Duncan AF. Comparison of the Bayley II mental developmental index and the Bayley III cognitive scale: Are we measuring the same thing? Acta Paediatr Int J Paediatr. 2012;101(2):55–8. 10.1111/j.1651-2227.2011.02517.x PMC3560971 22054168

[35] Squires J, Twombly E, Bricker D, Potter L. ASQ-3 Ages and stages questionnaires user’s guide. Lane County; 2009.

[36] Population by age and sex. China Statisitical Yearbook 2014.

[37] Simard M-N, Luu TM, Gosselin J. Concurrent validity of Ages and Stages Questionnaires in Preterm Infants. Pediatrics. 2012;130(1):e108–14. 10.1542/peds.2011-3532 22689873

[38] Martinez S, Naudeau S, Pereira V. The promise of preschool in Africa: A randomized impact evaluation of early childhood development in rural Mozambique. 2012.

[39] Velikonja T, Edbrooke-Childs J, Calderon A, Sleed M, Brown A, Deighton J. The psychometric properties of the Ages & Stages Questionnaires for ages 2–2.5: a systematic review. Child Care Health Dev. 2017;43(1):1–17. 10.1111/cch.12397 27554865

[40] Kwun Y, Park HW, Kim M ju, Lee BS, Kim EAR. Validity of the ages and stages questionnaires in Korean compared to bayley scales of infant development-II for screening preterm infants at corrected age of 18–24 months for neurodevelopmental delay. J Korean Med Sci. 2015;30(4):450–5. 10.3346/jkms.2015.30.4.450 25829813 PMC4366966

[41] Mackin R, Fadel N Ben, Feberova J, Murray L, Nair A, Kuehn S, et al ASQ3 and/or the bayley-III to support clinicians’ decision making. PLoS One. 2017;12(2):1–13. 10.1371/journal.pone.0170171 PMC5289417 28151969

[42] Ga H, Kwon JY. A comparison of the Korean-Ages and Stages Questionnaires and Denver Developmental Delay Screening Test. Ann Rehabil Med. 2011;35(3):369 10.5535/arm.2011.35.3.369 22506146 PMC3309214

[43] Bai Y, Zhang L, Liu C, Shi Y, Mo D, Rozelle S. Effect of parental migration on the academic performance of left behind children in north western China. J Dev Stud. 2018;54(7):1154–70.

[44] Carneiro P, Meghir C, Parey M. Maternal education, home environments, and the development of children and adolescents. J Eur Econ Assoc. 2013;

[45] Christian K, Morrison FJ, Bryant FB. Predicting kindergarten academic skills: Interactions among child care, maternal education, and family literacy environments. Early Child Res Q. 1998;

[46] Dollaghan CA, Campbell TF, Paradise JL, Feldman HM, Janosky JE, Pitcairn DN, et al Maternal education and measures of early speech and language. J Speech, Lang Hear Res. 2014; 10.1044/jslhr.4206.1432 10599625

[47] Yue A, Shi Y, Luo R, Wang B, Weber A, Medina A, et al Parental stimulation and early child development in China: Parenting at Arm’s Length. 2018. 10.1097/DBP.0000000000000678 31107768

[48] Hix-Small H, Marks K, Squires J, Nickel R. Impact of Implementing developmental screening at 12 and 24 months in a pediatric practice. Pediatrics. 2007;120(2):381–9. 10.1542/peds.2006-3583 17671065

[49] Fernandes M, Stein A, Newton CR, Cheikh-Ismail L, Kihara M, Wulff K, et al The INTERGROWTH-21st Project neurodevelopment package: A novel method for the multi-dimensional assessment of neurodevelopment in pre-school age children. PLoS One. 2014;9(11):e113360 10.1371/journal.pone.0113360 25423589 PMC4244160

[50] Care I, Alerts E. Neurodevelopment outcome in extremely preterm infants at 2.5 years after active perinatal care in Sweden. Jama. 2009;309(17):1810–20. 10.1001/jama.2013.3786 23632725

[51] Weiss L., Oakland T, Aylward G. Bayley-III clinical use and interpretation Academic Press 2010.

[52] Greubel L, Van Der Gaag J. Early childhood development: A Chinese national priority and global concern for 2015. The Brookings Institute 2012.

