# Supplementary material for: Concurrent validity of the Ages and Stages Questionnaire and the Bayley Scales of Infant Development III in China
Source: PLoS One. 2019 Sep 5;14(9):e0221675. doi: 10.1371/journal.pone.0221675 (PMC6728026; doi:10.1371/journal.pone.0221675)
Supplement: S1 Appendix — Table A. Characteristics of the Bayley-III and the ASQ-3 Table B. Correlations of the Bayley-III and the ASQ with Maternal Education, Household Wealth, Play Activities and Play Materials in the Home, All Ages Combines (Using Bayley scaled scores) Table C. Pass/fail agreement between the ASQ and Bayley (Mom is the primary caregiver) Table D. Pass/fail agreement between the ASQ and Bayley (Grandmother is the primary caregiver). (DOCX) [file pone.0221675.s001.docx]

|  | Age range test (mths) | Age range study children (mths) | Number of items | Time to administer (min) | Score range of the test | Cronbach’s alpha | |
| --- | --- | --- | --- | --- | --- | --- | --- |
| Criterion Measure |  |  |  |  |  |  |  |
| Bayley-III |  |  |  |  |  |  |  |
| Cognitive | 0-42 | 5-24 | 91 | 35 | 55-145 | 0.971 | 0.973 |
| Receptive Language | 0-42 | 5-24 | 48 | 15 | 47-153 | 0.962 | 0.966 |
| Expressive Language | 0-42 | 5-24 | 47 | 15 | 47-153 | 0.965 | 0.969 |
| Fine Motor | 0-42 | 5-24 | 66 | 20 | 46-154 | 0.955 | 0.960 |
| Gross Motor | 0-42 | 5-24 | 72 | 15 | 46-154 | 0.965 | 0.974 |
| ASQ-3 |  |  |  |  |  | Caregiver is grandmother | Caregiver is mother |
| Problem Solving | 1-66 | 5-24 | 6 | 3 | 0-60 | 0.600 | 0.672 |
| Communication | 1-66 | 5-24 | 6 | 3 | 0-60 | 0.640 | 0.595 |
| Fine Motor | 1-66 | 5-24 | 6 | 3 | 0-60 | 0.539 | 0.601 |
| Gross Motor | 1-66 | 5-24 | 6 | 3 | 0-60 | 0.712 | 0.719 |
| Personal-Social | 1-66 | 5-24 | 6 | 3 | 0-60 | 0.447 | 0.598 |

Table A. Characteristics of the Bayley-III and the ASQ-3

|  | **Maternal Education** | **Wealth Index** | **Play Acivities** | **Play Materials** |
| --- | --- | --- | --- | --- |
| **Bayley-III** |  |  |  |  |
| Cognitive | 0.060** | 0.058*** | 0.026 | 0.084*** |
| Receptive Language | 0.066*** | 0.068*** | 0.054*** | 0.073*** |
| Expressive Language | 0.068** | 0.072*** | 0.037* | 0.063*** |
| Fine Motor | 0.049* | 0.047*** | 0.045** | 0.082*** |
| Gross Motor | 0.008 | 0.046*** | 0.036** | 0.064*** |
| **ASQ-3** |  |  |  |  |
| Problem Solving | 1.142 | 1.705*** | 0.163*** | 0.251*** |
| Communication | 2.790** | 1.590*** | 0.215*** | 0.236*** |
| Fine Motor | 1.635 | 1.883*** | 0.176*** | 0.228*** |
| Gross Motor | -2.302 | 1.558*** | 0.147*** | 0.231*** |
| Personal-Social | 0.687 | 1.501*** | 0.161*** | 0.225*** |

Table B. Correlations of the Bayley-III and the ASQ with Maternal Education, Household Wealth, Play Activities and Play Materials in the Home, All Ages Combines (Using Bayley scaled scores)

Note: * p<0.05; ** p<0.01; *** p<0.001.

Table C. Pass/fail agreement between the ASQ and Bayley (Mom is the primary caregiver)

|  | Age cohorts | Domain | True positives (%) | False positives (%) | Ture negatives (%) | False negatives (%) | Sensitivity | Specificity |
| --- | --- | --- | --- | --- | --- | --- | --- | --- |
| 1SD | 5-12 months | cognitive | 0.199 | 0.194 | 0.413 | 0.194 | 50.6% | 68.0% |
|  |  | language | 0.28 | 0.129 | 0.27 | 0.321 | 46.6% | 67.7% |
|  |  | motor | 0.549 | 0.198 | 0.123 | 0.131 | 80.7% | 38.3% |
|  | 13-18 months | cognitive | 0.113 | 0.159 | 0.545 | 0.183 | 38.2% | 77.4% |
|  |  | language | 0.241 | 0.123 | 0.383 | 0.253 | 48.8% | 75.7% |
|  |  | motor | 0.164 | 0.349 | 0.448 | 0.039 | 80.8% | 56.2% |
|  | 19-24 months | cognitive | 0.065 | 0.072 | 0.502 | 0.362 | 15.2% | 87.5% |
|  |  | language | 0.085 | 0.055 | 0.529 | 0.331 | 20.4% | 90.6% |
|  |  | motor | 0.041 | 0.273 | 0.635 | 0.051 | 44.6% | 69.9% |
| 2SD | 5-12 months | cognitive | 0.03 | 0.178 | 0.734 | 0.058 | 34.1% | 80.5% |
|  |  | language | 0.03 | 0.113 | 0.707 | 0.15 | 16.7% | 86.2% |
|  |  | motor | 0.224 | 0.257 | 0.395 | 0.123 | 64.6% | 60.6% |
|  | 13-18 months | cognitive | 0.024 | 0.118 | 0.827 | 0.031 | 43.6% | 87.5% |
|  |  | language | 0.036 | 0.036 | 0.817 | 0.111 | 24.5% | 95.8% |
|  |  | motor | 0.041 | 0.243 | 0.704 | 0.012 | 77.4% | 74.3% |
|  | 19-24 months | cognitive | 0.003 | 0.038 | 0.932 | 0.027 | 10.0% | 96.1% |
|  |  | language | 0.017 | 0.034 | 0.898 | 0.051 | 25.0% | 96.4% |
|  |  | motor | 0.007 | 0.154 | 0.826 | 0.014 | 33.3% | 84.3% |

Table D. Pass/fail agreement between the ASQ and Bayley (Grandmother is the primary caregiver)

|  | Age cohorts | Domain | True positives (%) | False positives (%) | Ture negatives (%) | False negatives (%) | Sensitivity | Specificity |
| --- | --- | --- | --- | --- | --- | --- | --- | --- |
| 1SD | 5-12 months | cognitive | 0.174 | 0.357 | 0.339 | 0.130 | 57.2% | 48.7% |
|  |  | language | 0.322 | 0.165 | 0.200 | 0.313 | 50.7% | 54.8% |
|  |  | motor | 0.557 | 0.183 | 0.148 | 0.113 | 83.1% | 44.7% |
|  | 13-18 months | cognitive | 0.088 | 0.155 | 0.591 | 0.166 | 34.6% | 79.2% |
|  |  | language | 0.232 | 0.149 | 0.365 | 0.254 | 47.7% | 71.0% |
|  |  | motor | 0.149 | 0.398 | 0.420 | 0.033 | 81.9% | 51.3% |
|  | 19-24 months | cognitive | 0.123 | 0.086 | 0.405 | 0.387 | 24.1% | 82.5% |
|  |  | language | 0.184 | 0.055 | 0.479 | 0.282 | 39.5% | 89.7% |
|  |  | motor | 0.074 | 0.282 | 0.577 | 0.067 | 52.5% | 67.2% |
| 2SD | 5-12 months | cognitive | 0.043 | 0.217 | 0.704 | 0.035 | 55.1% | 76.4% |
|  |  | language | 0.061 | 0.165 | 0.591 | 0.183 | 25.0% | 78.2% |
|  |  | motor | 0.183 | 0.296 | 0.374 | 0.148 | 55.3% | 55.8% |
|  | 13-18 months | cognitive | 0.011 | 0.122 | 0.834 | 0.033 | 25.0% | 87.2% |
|  |  | language | 0.028 | 0.033 | 0.823 | 0.116 | 19.4% | 96.1% |
|  |  | motor | 0.017 | 0.221 | 0.740 | 0.022 | 43.6% | 77.0% |
|  | 19-24 months | cognitive | 0.018 | 0.080 | 0.847 | 0.055 | 24.7% | 91.4% |
|  |  | language | 0.037 | 0.074 | 0.828 | 0.061 | 37.8% | 91.8% |
|  |  | motor | 0.012 | 0.166 | 0.804 | 0.018 | 40.0% | 82.9% |
